# Single-Vesicle Molecular Profiling by dSTORM Imaging in a Liquid Biopsy Assay Predicts Early Relapse in Colorectal Cancer

**DOI:** 10.3390/biom15091307

**Published:** 2025-09-11

**Authors:** Gabriele Raciti, Giulia Cavallaro, Raffaella Giuffrida, Cristina Grange, Loredana Leggio, Marco Catania, Nunzio Iraci, Elena Bruno, Luca Antonio Giaimi, Sofia Paola Lombardo, Giulia Chisari, Marzia Mare, Enrica Deiana, Lorenzo Memeo, Benedetta Bussolati, Stefano Forte

**Affiliations:** 1 Mediterranean Institute of Oncology, 95029 Catania, Italy; gabriele.raciti@grupposamed.com (G.R.); giulia.cavallaro@grupposamed.com (G.C.); raffaella.giuffrida@grupposamed.com (R.G.); luca.giaimi@grupposamed.com (L.A.G.); sofia.lombardo@grupposamed.com (S.P.L.); giulia.chisari@grupposamed.com (G.C.); marzia.mare@grupposamed.com (M.M.); enrica.deiana@grupposamed.com (E.D.); lorenzo.memeo@grupposamed.com (L.M.); 2 Department of Medical Sciences, University of Turin, 10124 Turin, Italy; cristina.grange@unito.it; 3 Department of Biomedical and Biotechnological Sciences, University of Catania, 95123 Catania, Italy; loredana.leggio@unict.it (L.L.); marco.catania@phd.unict.it (M.C.); nunzio.iraci@unict.it (N.I.); 4 Department of Physics and Astronomy ‘Ettore Majorana’, University of Catania, Via S. Sofia 64, 95123 Catania, Italy; elena.bruno@dfa.unict.it; 5 Consiglio Nazionale delle Ricerche, Istituto per la Microelettronica e i Microsistemi (CNR-IMM), Catania (University) Unit, Via S. Sofia 64, 95123 Catania, Italy; 6 Department of Molecular Biotechnology and Health Sciences, University of Turin, 10124 Turin, Italy; benedetta.bussolati@unito.it

**Keywords:** colorectal cancer, liquid biopsy, extracellular vesicles, super-resolution microscopy (dSTORM), protein biomarkers

## Abstract

Background and Objectives: Colorectal cancer (CRC) is the third most diagnosed tumor type and the second leading cause of cancer-related mortality. Despite recent improvements in the clinical management of CRC patients both before and after surgery, disease recurrence remains common, with an incidence of about 20–30% within 5 years. Current tissue biopsy techniques are invasive and inadequate for assessing tumor heterogeneity or capturing real-time disease dynamics. In contrast, liquid biopsy offers a promising, minimally invasive alternative. This study aimed to evaluate extracellular vesicle (EV)-associated protein markers, detected through super-resolution microscopy, as potential indicators of recurrence in CRC patients. Methods: We employed a novel liquid biopsy approach based on the super-resolution imaging (dSTORM) of specific protein markers carried by EVs isolated from the plasma of CRC patients. We analyzed combinations of both surface and intravesicular proteins, including EpCAM, PD-L1, CD81, IL-6, and Cyclin D1. Results: Specific combinations of EV-associated markers were able to distinguish patients with recurrence from those without residual disease. Additionally, we observed correlations between some marker profiles and tumor stage or lymph node involvement. No association was found with mismatch repair system status. Conclusions: To our knowledge, this is the first study to propose the use of EV-bound proteins for recurrence detection in CRC using super-resolution microscopy within a liquid biopsy framework. These findings support the potential of this approach as a non-invasive tool for CRC monitoring.

## 1. Introduction

Although the cancer mortality rate is overall decreasing worldwide, cancer still represents the second cause of death after cardiovascular diseases [1]. In fact, 28.4 million new cancer cases are estimated to occur in 2040, almost doubling the number of cases in 2020 [2]. Colorectal cancer (CRC) currently ranks as the third most diagnosed tumor type, preceded only by breast and lung cancers, and it ranks second in terms of mortality [3]. In recent decades, its incidence varied according to the developmental status of countries, showing an increase in low- and medium-Human Development Index (HDI) countries and a slight, but steady, decrease (especially in mortality) in countries with a high HDI [4]. In CRC patients, tissue biopsy and radiographic imaging are performed for diagnostic purposes, and the mutational status of specific genes, including KRAS, NRAS, and BRAF, is tested in order to personalize the treatment regimen [5]. Nevertheless, tissue biopsy has an intrinsic limit in terms of providing a snapshot of the CRC molecular landscape, underestimating cancer spatial and temporal heterogeneity. It is known that tumor heterogeneity can determine the evolution of different tumor subclones under the pressure of therapy, leading to long-term pharmacological resistance mechanisms [6,7,8]. Moreover, repetitive tissue samplings are not conceivable because of the invasiveness of the technique. Despite the improvement in CRC patients’ clinical management, recurrence is still widespread, with an incidence of about 20–30% within 5 years after primary resection and mostly within 2 years [9,10]. Several factors affect the possibility of relapse in stage I-III CRC patients: age, localization of the primary tumor, staging, positive lymph node status, tumor budding, presence of metastasis, positive surgical margins, and adjuvant treatment after surgery or not [11,12,13,14]. Hence, a post-operative follow-up is needed to avoid the late detection of tumor relapse.

In recent years, researchers have sought to replace standard and invasive methods of profiling tumors with liquid biopsy, which presents several important advantages, like ease of sampling, minimal invasiveness, possibility to temporarily monitor cancer course, and repeatability. Tumor-derived entities, like circulating tumor cells (CTCs), circulating tumor DNA, and tumor extracellular vesicles (EVs), are shed throughout the body fluids, although mostly in the blood. Therefore, genomic and proteomic analyses of these factors can give important hints for prognosis, tumor follow-up, and response to therapy [15]. In particular, EVs, whose protected cargo mirrors the state of the parental cells, have become a paramount tool in liquid biopsy for their role in cell-to-cell communication, being key players in tumor growth, epithelial-to-mesenchymal transition, metastasis, immunosuppression, etc. [16].

EpCAM, PD-L1, Cyclin D1, and IL-6 are among the most investigated proteins, even though there is uncertain evidence about their roles in CRC. Low/null epithelial cell adhesion molecule (EpCAM) tissue expression makes cells prone to dissociating from each other and to disseminating from tumor bulk. In fact, its decreased expression worsens the pathological scenario with the overall worst prognosis in CRC patients [17,18]. Furthermore, Lynch syndrome has been linked with germinal EpCAM-inactivating mutations [19]. The role of Programmed cell death 1 ligand 1 (PD-L1) in CRC management is controversial: only a minority of CRC individuals (typically MSI ones) positively react to anti-PD-L1/PD-1 therapy [20]. Moreover, its role as a prognostic factor is questionable: high circulating levels pre-treatment have been associated with locally advanced and potentially invasive tumors [21,22], while post-treatment detection might anticipate an unfavorable prognosis [23] or predict response and clinical benefit [24]. Cyclin D1 has been found to be overexpressed in CRC tumor samples, but meta-analyses revealed uncertain results [25]. Thereafter, its prognostic value was debated, and it has swung between being a good and bad prognostic indicator [25,26], showing either a correlation with poor overall survival and the worst relapse rate [27] or no correlation [28]. The involvement of interleukins in CRC has been well documented [29]; indeed, high levels of interleukin 6 (IL-6) seem to be associated with the worst disease outcome [30,31]. In addition, some authors have underlined the potential of IL-6 as a prognostic marker given its association with survival and relapse rates [32,33]. Moreover, Tseng-Rogenski et al. have speculated about a possible relationship between IL-6 signaling and mismatch repair system dysfunctions [34]. The tetraspanin CD81 would act as a tumor suppressor in some cancers [35,36] or as a tumor-promoting factor in others [37,38,39,40,41]. Furthermore, its gene downregulation or knocking out would favor an invasive phenotype both in vitro and in vivo [42]. Thus, this evidence requires further investigations about the role of these markers in the CRC context.

In this work, our group investigated the feasibility of a blood-based liquid biopsy approach to the prediction of the prognosis of CRC. In particular, this method relies on the evaluation of specific blood-derived small extracellular vesicle (sEV) protein markers assessed by super-resolution microscopy. This technique, previously described for distinguishing prostate cancer patients from individuals with hyperplasia conditions [43], has shown to be reproducible and sensitive enough to discriminate CRC patients likely to experience relapse from ones without any residual disease. Furthermore, we showed a correlation between cluster count levels and CRC patients’ clinical features. Finally, we tested the possibility of classifying a subset of CRC patients on the basis of their mismatch repair system status.

## 2. Materials and Methods

### 2.1. Patients’ Enrollment, Blood Sampling, and Plasma Isolation

This study was conducted in accordance with the Declaration of Helsinki and approved by the Ethics Committee CATANIA2 of Azienda Ospedaliera di Rilievo Nazionale e di Alta Specializzazione “Garibaldi” (protocol code 436 of 20 July 2020). Informed consent was obtained from all the subjects involved in this study.

The inclusion criteria for patient enrolment were as follows: age ≥ 18 years, a diagnosis of stage II-III CRC, eligibility for surgical resection followed by adjuvant chemotherapy with FOLFOX or XELOX/CAPOX, and a SARS-CoV-2-negative status. Exclusion criteria included patients with malignancies other than CRC, those ineligible for adjuvant chemotherapy, individuals with viral infections or other conditions that could interfere with the results, and pregnant women.

For each enrolled CRC patient, a pre-surgical blood sample (10 mL) was collected. The blood samples were immediately processed for plasma separation. Briefly, the samples were centrifuged at 1600× *g* for 10 min at room temperature (RT) to remove the cellular components. The resulting supernatant (plasma) was then subjected to a second centrifugation step at 3000× *g* for 10 min to eliminate any remaining cell debris potentially carried over from the previous step. Finally, the recovered plasma was aliquoted into 1 mL tubes and stored at −80 °C for subsequent analyses.

### 2.2. Mismatch Repair Status Evaluation

MMR status was assessed on tumor tissue samples using immunohistochemistry (IHC) for a panel of four key proteins. Specifically, the expression of MSH2, MSH6, MLH1, and PMS2 was evaluated according to national diagnostic guidelines for the identification of mismatch repair (MMR) deficiency. IHC staining was performed using the fully automated Roche Ventana Benchmark Ultra with Roche Ventana antibodies MLH1 (clone M1), MSH2 (clone G219-1129), MSH6 (clone 44), and PMS2 (clone EPR3947). The loss of nuclear staining in tumor cells, in the presence of intact expression in surrounding non-tumor cells, was interpreted as indicative of deficient MMR (dMMR) status.

### 2.3. Isolation of Extracellular Vesicles

The isolation of extracellular vesicles was performed as follows: First, 2 mL aliquots of frozen plasma samples were rapidly thawed at 37 °C and then immediately placed on ice. The samples were subjected to sequential centrifugation conducted at 4 °C. The first centrifugation was performed for 10 min at 4000 rpm to remove any residual cells; the second one was conducted for 30 min at 10,500 rpm to eliminate cellular debris and large EVs. Before proceeding with ultracentrifugation (UC) to isolate small EVs (sEVs), the plasma was purified by passaging through a 0.22 µm filter. Ultracentrifugation was carried out for 60 min at 200,000× *g*, in a Sorvall Discovery 90SE ultracentrifuge equipped with a Thermo Scientific (Waltham, MA, USA) TH-660 Titanium swinging bucket rotor. Subsequently, the sEVs were washed with PBS 1× and subsequently subjected to another ultracentrifugation step. The resulting pellet was resuspended in 100 µL of cold PBS and stored at −80 °C. For the quantification of sEV-associated proteins, 10 µL of the EV suspension was used according to the Pierce BCA protein assay kit (Thermo Scientific) protocol, and the protein concentration was determined using a BSA standard curve. Absorbance was measured spectrophotometrically at 562 nm.

### 2.4. Characterization of EVs

#### 2.4.1. Nanoparticle Tracking Analysis

EV size and concentration were determined using the NanoSight Pro (Malvern Panalytical, Malvern, UK) fitted with a high-sensitivity sCMOS (USB-3) camera and a 532 nm laser. EV samples were diluted in PBS 1× to give ~40 particles per frame. Camera exposure, gain, and focus were set as automatic, and six 750-frame videos were then acquired and analyzed with a raw distribution type.

#### 2.4.2. Scanning Electron Microscopy (SEM)

EV morphology was studied using a Gemini Field Emission SEM Carl Zeiss SUPRATM 25 scanning electron microscope (FEG-SEM, Carl Zeiss Microscopy GmbH, Jena, Germany) in Inlens mode using a 3 kV electron beam. To prepare SEM samples, the EVs recovered from the 200,000× *g* pellet were first resuspended in 500 μL di H_2_O, and then a 50 μL aliquot of EV suspension was dropped onto pin stubs coated with PELCO carbon conductive tabs and left to dry at RT.

#### 2.4.3. Western Blot Analysis

Jurkat cells and EV extracts were processed as in [43,44]. Briefly, cells and EVs were lysed in RIPA buffer (10 mM Tris HCl pH 7.2 (Fisher Scientific, Waltham, MA, USA, BP152); 150 mM NaCl (Sigma Aldrich, Saint Louis, MI, USA, S7653); 1% Sodium deoxycholate (Sigma Aldrich, 30970); 0.1% (for cells) or 3% (for EVs) SDS (Sigma Aldrich, 71736); 1% Triton X-100 (Sigma Aldrich, T8787); 1 mM EDTA pH 8 (VWR chemicals, Radnor, PA, USA, E177-100ML); 1X Complete Protease inhibitor cocktail (Roche, Basel, Switzerland, 04693116001); and 1 mM Phenylmethanesulfonyl fluoride solution (PMSF, Sigma Aldrich, 93482). Protein concentration was measured with DC Protein Assay (Biorad, Hercules, CA, USA, 500-0116), using BSA (Pierce, Waltham, MA, USA, 23210) as the standard. A total of 17.5 µg of Jurkat cell lysate and 30 µg of EV lysates were loaded into 4–12% Bis-Tris plus gels (Invitrogen, Waltham, MA, USA, NW04125BOX) under reducing conditions, while 15 µg of EV lysates was used for not reducing conditions. Proteins were transferred onto a PVDF membrane. All primary and secondary antibodies are listed in Table 1. Jurkat cells were used as the positive control for CD45.

#### 2.4.4. Super-Resolution Microscopy

To analyze the extracellular vesicle samples, three-dimensional (3D) direct stochastic optical reconstruction microscopy (dSTORM) was employed, using the Nanoimager S Mark II microscope from ONI (Oxford Nanoimaging, Oxford, UK). This microscope is equipped with a 100×, 1.4 NA oil immersion objective, an XYZ closed-loop piezo 736 stage, and triple emission channels split at 473/488 nm, 561 nm, and 640 nm. The system employs a factory-configured optical path with pre-installed excitation and emission filters for these channels; these components are not user-adjustable and were used without modification. Illumination time was set to 30 milliseconds per frame, while 55 mW and 120 mW were chosen as laser power parameters for dyes excited with the 560/640 nm and 488 nm wavelengths, respectively, in accordance with the manufacturer’s indication.

All experiments were conducted using the EV Profiler Kit from ONI (product code: EV-MAN-1.0, Oxford Nanoimaging, Oxford, UK) and performed at RT. In brief, as reported in the manufacturer’s protocol, the assay chip surface was activated by introducing 5 µL of Surface Solution S3 in each of the four lanes, following a 10 min incubation. After washing with W1 Wash Solution to remove the excess S3, 10 µL of S4 Surface Solution was pipetted into each lane and incubated for an additional 10 min.

The EV capture process was performed as follows: First, 6 µL of sEV suspension was added to a mixture consisting of 3 µL of W1 and 1 µL of C1 Capture Supplement. This mixture was then distributed onto the lane surfaces and incubated for 30 min to capture the EVs. To remove unbound sEVs, a washing step with W1 was performed, followed by a 10 min incubation with 20 µL of F1 Fixation Solution.

After washing, each lane underwent a 10 min incubation with 10 µL of P1 solution (for permeabilization and blocking) to facilitate the staining of intravesicular targets. As per recommendations, an initial working solution with a 1:20 dilution of each antibody in W1 was prepared, and 1 µL of this solution was added to 9 µL of either P1 or N1 solution to achieve a final dilution of 1:200. Subsequently, 10 µL of the antibody solution was added to each lane and incubated for 50 min, protecting the chip from light.

Following another washing step, 20 µL of F1 was applied and incubated for 10 min. Finally, 50 µL of BCubed imaging buffer was added to each lane, and image acquisition was immediately started.

Three channels (640 nm, 561 nm, and 473/488 nm) of dSTORM data were sequentially acquired at 30 Hz in total reflection fluorescence (TIRF) mode, with at least three acquisitions for each sample. Before each imaging session, to align fluorescent channels, to ensure a channel mapping precision smaller than 12 nm, and to minimize chromatic aberrations, calibration was performed using beads on a slide supplied by the company. Moreover, to minimize differences in blinking kinetics across dyes, samples were processed with the ONI imaging buffer optimized for multicolor dSTORM. Repeated blinking events from the same molecule were consolidated by the software to avoid overcounting. Localizations with a low signal-to-noise ratio or not associated with a vesicle region of interest (ROI) were excluded. Each extracellular vesicle was identified as an ROI on the functionalized coverslip. An EV was considered positive for a given marker when at least one cluster of that marker was detected within its ROI. Accordingly, EVs were classified as single-, double-, or triple-positive when clusters from one, two, or three markers, respectively, were simultaneously present in the same vesicle.

All single-molecule localization data were analyzed using algorithms developed by the ONI company via their CODI website platform (https://alto.codi.bio/, accessed on 22 May 2023) to minimize background noise and remove low-precision and non-specific co-localization. A cluster was defined as the localizations from the dSTORM image which corresponds to each of the proteins labeled on the EV surface, in accordance with the official validated technical and methodological information and guidelines provided by the technology developer (Oxford Nanoimaging). All localizations within a defined radius are grouped into a dense circular cluster representing a single EV. Once these clusters have been identified, they can be constrained on parameters such as size, shape, length, and circularity. According to this definition, the terms EV and cluster are synonymous (although not technically) for the purpose of the present work.

For the internal validation of the clustering analysis, canonical tetraspanins (e.g., CD81), which are well documented to form nanodomains on EV membranes, were analyzed alongside IL-6, a soluble cargo protein expected to display a more diffuse distribution. This provided a biologically grounded reference for clustered versus heterogeneous patterns. All three fluorophores (AF^®^488, AF^®^555, CF^®^647) were imaged under identical acquisition settings.

sEV population phenotypes (triple-, double-, or single-positive for each channel otherwise to be intended for vesicles labeled for three, two, or one of the used antibodies) and the number of clusters for each phenotype were determined and analyzed separately.

All described reagents were supplied in the kit, including fluorescent antibodies anti-CD9-CF^®^488A, anti-CD63-CF^®^568, and anti-CD81-CF^®^647. P1 buffer was kindly supplied separately by ONI. Additionally, custom antibodies anti-PD-L1-AF^®^488 (Abcam, Cambridge Biomedical Campus, Cambridge, UK, ab237402), anti-EpCAM/CD326-AF^®^555 (Bioss Antibodies, Woburn, MA, USA, bs-1513R-A555), anti-IL-6-AF^®^555 (Bioss Antibodies, Woburn, MA, USA, bs-4587R-A555), and anti-Cyclin D1-AF^®^488 (Bioss Antibodies, Woburn, MA, USA, bs-0623R-A488) were used.

### 2.5. Statistical Analysis

Statistical analyses were performed on the per-sample counts of positive EVs, which were log2-transformed prior to group comparisons to reduce data range and improve distribution symmetry. Welch’s *t*-test was employed to compare intersample means. To assess the classification performance of biomarkers, contingency tables and receiver operating characteristic (ROC) curve analyses were performed. For multiple testing correction in post hoc analyses, the Benjamini–Hochberg method was applied. The results were considered statistically significant when the *p*-value or the adjusted *p*-value (*p*-adj) was less than 0.05. All analyses were conducted using R (v4.3.0) [45] and RStudio (v2023.6.0 Build 421) [46]. Data visualization was carried out with the ggpubr [47] and ggplot2 [48] packages, while ROC analyses were performed using the ROCR package [49]. Both individual and combined intravesicular or surface targets were statistically compared.

## 3. Results

### 3.1. Characterization of sEVs

EV pellets from a representative subset of pre-operative sampling-derived plasma of the 33 CRC subjects were first characterized via Nanoparticle Tracking Analysis (NTA) and Scanning Electron Microscopy (SEM) for size and concentration and then with the Western Blot (WB) technique to ensure the presence of specific sEV markers as well as the absence of blood cell-derived contaminants. All enrolled patients’ clinical characteristics are summarized in Table 2.

**Figure 1:**
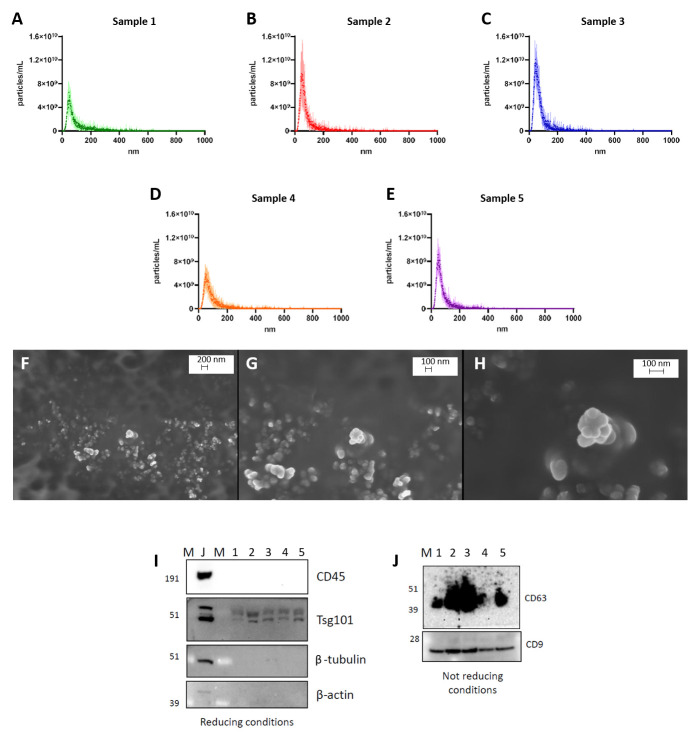
Characterization of EVs. NTA of five representative sEV samples isolated from CRC patients (**A**–**E**). Representative images of sEV acquisitions by SEM at different magnifications (**F**–**H**). Western Blot analysis on Jurkat (**J**) and vesicular lysates (1, 2, 3, 4, and 5) to detect CD45, Tsg101, β-tubulin, and β-actin in reducing conditions and CD63 and CD9 in not reducing ones (**I**,**J**). Abbreviations: NTA: Nanoparticle Tracking Analysis; sEV: small extracellular vesicle; CRC: colorectal cancer; SEM: Scanning Electron Microscopy; J: Jurkat cell lysate; M: marker.

NTA on five representative plasma-derived EV samples confirmed an enrichment in small vesicle populations isolated through ultracentrifugation. Isolated EVs exhibited an average size of approximately 90 nm and an average concentration around 4.5 × 10^11^/mL (Figure 1A–E, Supplementary Table S1). Furthermore, the morphology and size of isolated nanoparticles were assessed by SEM, revealing a moderate level of homogeneity for both parameters (Figure 1F–H). All the samples, in fact, exhibited an enrichment in vesicle populations, with an average size of approximately 100 nm, confirming NTA data.

Additionally, WB analysis further supported the pellet’s vesicular nature. In fact, the samples displayed the presence of both intravesicular markers such as Tsg101, an ESCRT-1 component, and surface ones, like the CD9 and CD63 tetraspanins. Also, the absence of CD45 in the EV lysates underlined the absence of hematopoietic contamination that might arise from blood EV isolation. On the contrary, as expected, CD45 is significantly expressed in Jurkat cell lysate, an immortalized line of human T lymphocyte cells, together with β-tubulin and β-actin, both cytoskeletal components (Figure 1I,J, Supplementary Figure S1).

**Figure 2:**
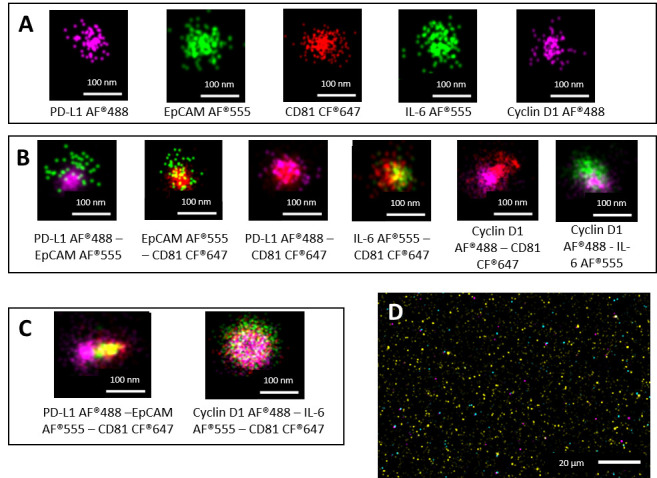
Super-resolution microscopy EV characterization. Representative images of single sEVs presenting only one (single labeling with scale bar of 100 nm) of analyzed markers (**A**) and coexpressing two (**B**) or three (**C**) markers contemporarily (double and triple labeling, respectively, with scale bar of 100 nm). Super-resolution microscopy field of view of sEVs isolated from CRC plasma sample with scale bar of 20 µm (**D**). Abbreviations: sEVs: small extracellular vesicles; CRC: colorectal cancer; AF: Alexa fluor dye; CF: cyanine-based far red fluorescent dye.

A further EV characterization was performed by super-resolution microscopy, in order to evaluate the presence of a small group of selected proteins, both surface (EpCAM, PD-L1 and CD81) and luminal (IL-6, Cyclin D1). Notably, they were quantitatively compared according to patients’ tumor recurrence status and tumor clinical features and with their mismatch repair system status. Figure 2 shows a panel of representative images, derived by dSTORM acquisitions, of single EVs presenting only one (Figure 2A), two (Figure 2B), or three (Figure 2C) of the analyzed markers (single, double, and triple labeling, respectively).

### 3.2. Selected Vesicular Markers Correlate with Poor Prognosis in Colorectal Cancer Patients

**Figure 3:**
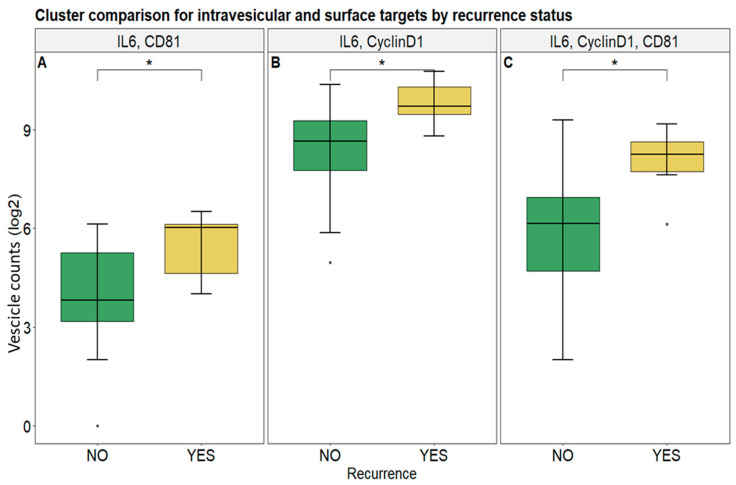
Cluster count comparison between patients with and without recurrence for selected intravesicular and surface targets. Subfigures represent box plots of vesicles presenting IL-6 and CD81 (**A**), IL-6 and Cyclin D1 (**B**), and IL-6, Cyclin D1, and CD81 in combination (**C**). Symbols indicate “*” *p*-adj < 0.05; “•” value out of ±1.5× IQR.

A quantitative comparison was performed to assess the number of clusters presenting intravesicular and surface markers, either individually or in combination. Note that the term “cluster” can be considered, although not technically, synonymous to sEV according to official validated technical and methodological information (see Section 2 for more information). CD81, IL-6, and Cyclin D1 values were found to be significantly higher in patients who experienced disease recurrence compared to those who did not (*p*-adj < 0.05), indicating their potential role as prognostic biomarkers for recurrence (Figure 3). In fact, the combinations of IL-6 and CD81, IL-6 and Cyclin D1, and IL-6, Cyclin D1, and CD81 as triplets showed significantly higher levels in recurrent cases, further supporting their relevance in predicting disease recurrence. However, clusters presenting only IL-6 or Cyclin D1, as well as the double-positive CD81/Cyclin D1 ones, showed no significant differences (Supplementary Figure S2B,C,H).

Likewise, surface proteins such as PD-L1, EpCAM, and CD81, both alone and when combined with each other as double- and triple-positive clusters, did not show statistically significant differences between the two groups (Supplementary Figure S2A,D–G,I,J). 

**Figure 4:**
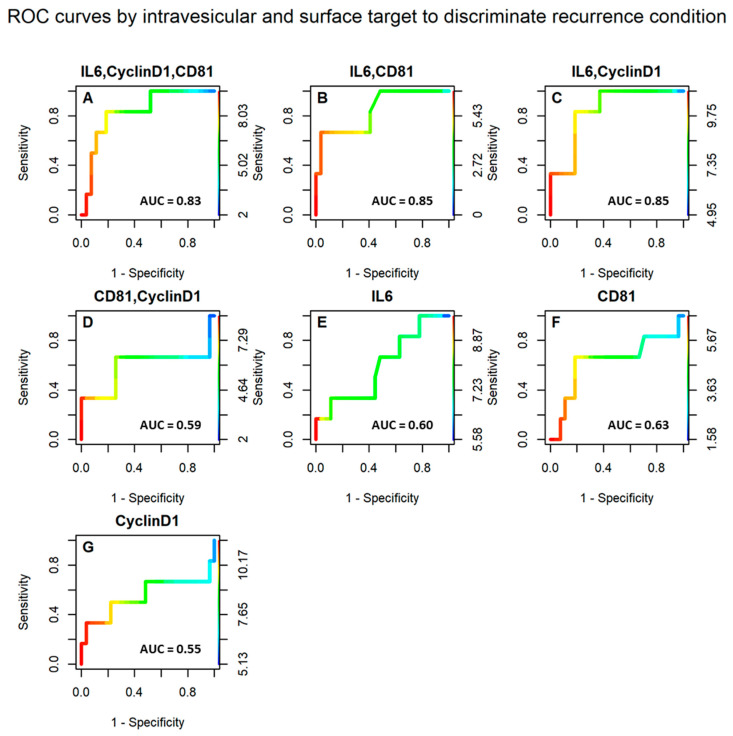
The receiver operating characteristic (ROC) curves for the intravesicular and surface targets. The ROC curves of IL-6, Cyclin D1, and CD81 (**A**); IL-6 and CD81 (**B**); IL-6 and Cyclin D1 (**C**); CD81 and Cyclin D1 (**D**); IL-6 (**E**); CD81 (**F**); and Cyclin D1 (**G**).

Moreover, the discriminatory capacity of the sEV markers to distinguish patients who experienced tumor recurrence from those who did not was further assessed through ROC curve analysis (Figure 4). The populations of clusters characterized by IL-6 and CD81 and the subgroup IL-6 and Cyclin D1 exhibited the highest area under the curve (AUC), with a value of 0.85 for both (Figure 4B,C). Another significant AUC value (0.83) was obtained for the combination of clusters positive for IL-6, Cyclin D1, and CD81 (Figure 4A). All other markers (CD81 alone, Cyclin D1 alone, IL-6 alone, and the CD81–Cyclin D1 group) showed a weaker ability to discriminate patients with respect to recurrence, with AUC values ranging from 0.55 to 0.63 (Figure 4D–G).

Therefore, the combination of specific intravesicular and surface EV markers holds significant discriminative power for recurrence status. Moreover, the predictive capacity of other surface markers was also evaluated; however, the results were not satisfactory (Supplementary Figure S3 and Table S2).

Table 3 displays the contingency tables for sEV markers and their performance metrics, derived by selected cutoff values. A detailed analysis of these results highlights that the combination of IL-6, Cyclin D1, and CD81 demonstrates the highest discriminatory power in differentiating patients who experience recurrence from those who do not, achieving an accuracy of 0.79. Similarly, the combination of IL-6 and Cyclin D1 also shows strong predictive capability, underscoring the potential of these markers as reliable indicators of recurrence risk.

### 3.3. Correlation of Selected Vesicular Markers with CRC Patients’ Tumor Clinical Features

**Figure 5:**
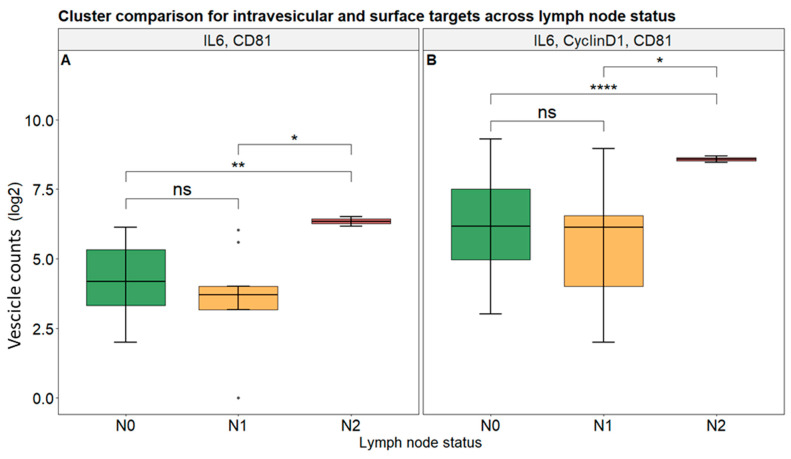
Cluster counts among lymph node (N) status for intravesicular and surface targets. Box plot analysis of vesicles presenting IL-6 and CD81 (**A**) and IL-6, Cyclin D1, and CD81 (**B**) in patients according to their different N statuses. Symbols represent “*” *p*-adj < 0.05; “**” *p*-adj < 0.01; “****” *p*-adj < 0.001; “ns” *p*-adj ≥ 0.05; “•” value out of ±1.5× IQR.

Next, we investigated the association of sEV markers with patients’ tumor clinical features. At first, we compared the cluster counts of all selected markers between patients without (N0) and with (N+) lymph node involvement, and no statistical significance arose from the analysis (Supplementary Figures S4 and S5). However, by dividing the N+ category in N1 and N2, we observed that both the combination of IL-6 and CD81 and the group comprising IL-6, Cyclin D1, and CD81 showed higher values in patients with advanced lymph node involvement (N2) compared to those with N0 or N1 status (*p*-adj < 0.05), even though the first ones are numerically low compared to the others. Thus, such data further support their association with both recurrence risk and lymphatic disease progression (Figure 5A,B).

**Figure 6:**
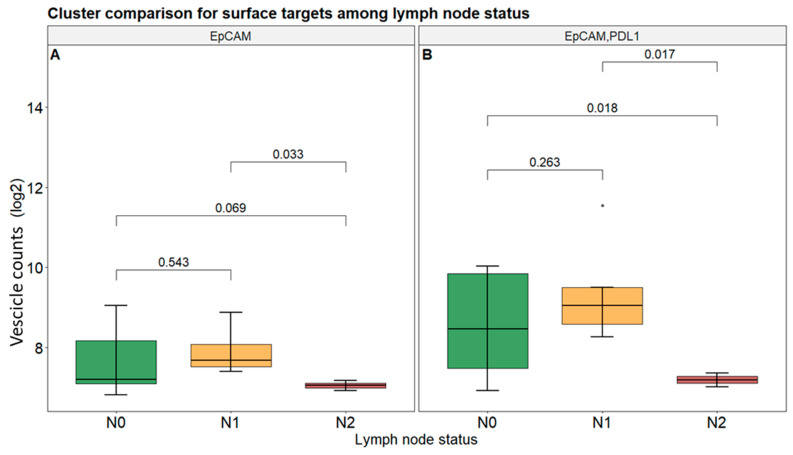
Cluster counts among lymph node (N) status for surface targets. Box plot analysis of vesicles presenting EpCAM (**A**) and EpCAM with PD-L1 (**B**) in patients according to their different N statuses. Statistical significance is assessed using *p*-value. Symbols represent “•” value out of ±1.5× IQR.

In addition, regarding the surface targets, EpCAM showed a statistically significant difference (*p* value < 0.05) between N1 and N2 status (Figure 6A). Also, EpCAM and PD-L1 as double-positive clusters displayed significantly lower values in N2 status compared to N0 and N1 (Figure 6B). This suggests that lower values of surface targets could be associated with lymph node involvement. All other combinations instead did not show any statistically significant difference (Supplementary Figure S6).

**Figure 7:**
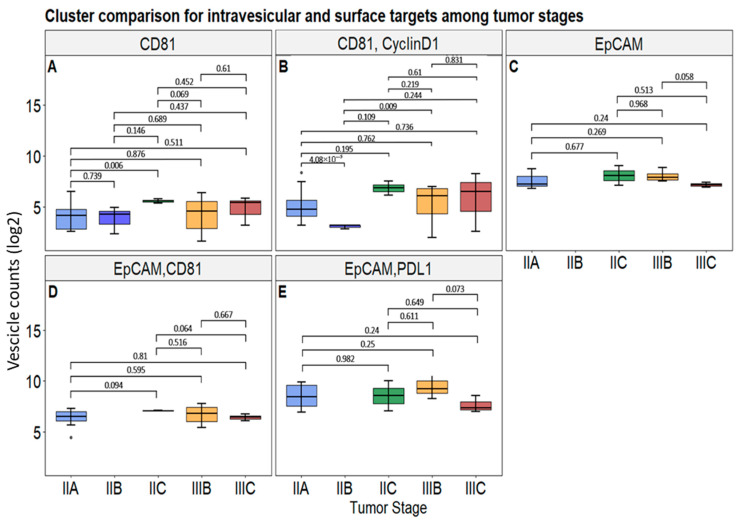
Cluster counts among tumor stages for the selected intravesicular and surface markers. Subfigures represent vesicles presenting CD81 (**A**), CD81 and Cyclin D1 (**B**), EpCAM (**C**), EpCAM and CD81 (**D**), and EpCAM and PD-L1 (**E**). The reported *p*-values are considered statistically significant when *p* < 0.05. Symbols represent “•” value out of ±1.5× IQR.

Cluster count differences were also analyzed according to tumor stages. As shown in Figure 7A,B, CD81-positive cluster counts were higher in stage IIC compared to IIA, and the combination of CD81 with Cyclin D1 showed an increased value in stage IIA relative to stage IIB. Moreover, a statistically significant difference was observed between stage IIB and stage IIIB, with higher levels in the latter. To note, among surface markers, both EpCAM alone and in combination with PD-L1 exhibited a tendency to discriminate between stages IIIB and IIIC, not reaching statistical significance though (Figure 7C,E). Likewise, the same tendency was observed in EpCAM/CD81 double-positive clusters regarding the IIC and IIIC stages, without being statistically significant either (Figure 7D). No other marker combinations showed any statistically significant differences by subgrouping stage status (Supplementary Figure S7) nor by grouping these categories into stage II and stage III overall (Supplementary Figures S8 and S9).

Lastly, we investigated the potential role of sEV-associated markers according to patients’ MMR status, by assessing differences between MMR-deficient and MMR-proficient groups (Supplementary Figure S10). However, they did not demonstrate any significant association with MMR status, thereby excluding their role as potential predictive biomarkers.

## 4. Discussion

Liquid biopsy is emerging as a promising technology progressively replacing more invasive and less reliable tissue biopsy. EVs released from cancer cells to the blood stream reflect the molecular composition of parental cells as vehicles for cancer markers, playing key roles in tumor progression. This specific capacity makes EVs of relevant clinical interest for diagnostic and prognostic purposes.

In this paper, we show that a combination of sEV-bounded protein markers allows us to discriminate patients who are likely to experience CRC relapse from those without any residual disease after surgery, exploiting an innovative technology (dSTORM). In addition, sEV markers correlate with tumor clinical features like tumor stage and lymph node status.

Direct stochastic optical reconstruction microscopy (dSTORM) overcomes the resolution limit of traditional optical microscopy (200–300 nm) [50,51]. With an imaging resolution of approximately 20 nm, in fact, it allows us to more deeply investigate subcellular architectures and intracellular pathway routes, prompting tremendous potential applications in biology and biotechnology. Hence, it has gained much attention among fluorescence-based microscopy techniques [52].

However, as reported in the latest MISEV guidelines [53], dSTORM has only been used to characterize EVs so far [54]. Despite its plasticity, only three groups have successfully applied such a technique in different experimental settings, beyond the detection of tetraspanins CD9, CD63, and CD81. Notably, Maire and colleagues demonstrated that DNA, protected and delivered inside glioblastoma cell-derived EVs, might allow for comprehensive methylation profiling and glioma subtype classification [55]. Verta et al., instead, demonstrated the feasibility of characterizing EVs presenting the SARS-CoV-2 spike protein through super-resolution microscopy and using them as a model for studying host–virus interactions, developing new therapeutic strategies [56]. Finally, our group has recently shown that vesicular protein combinations allow us to significantly discriminate patients with prostate cancer from individuals with hyperplasia [43]. In the present work, we demonstrated that by using this innovative technology, it is possible to detect EV clusters with specific combinations of surface and intravesicular markers. In particular, we found that IL-6/CD81, IL-6/Cyclin D1, and IL-6/Cyclin D1/CD81 as triplets were significantly more abundant in CRC recurrent cases compared to samples with no relapse (AUC: 0.85, 0.85, and 0.83, respectively). These data were further supported by the correlation with patients’ clinical features, including lymph node status and tumor grade. Indeed, clusters presenting the combinations IL-6/CD81 and IL-6/Cyclin D1/CD81 were significantly more expressed in patients with advanced lymph node involvement (N2) compared to those with N0 or N1 status. Similarly, even though less significant, cluster count differences were also found in correlation with tumor stages, further supporting their relevance in predicting disease recurrence.

CRC is a molecularly heterogeneous disease, and nearly 15% of its cases are caused by a pathological genetic condition due to the DNA mismatch repair (MMR) system, which normally corrects base pair mismatches during DNA replication, deficiency known as microsatellite instability (MSI), and a molecular phenotype characterized by length alterations in these repetitive sequences [57,58].

Tumors exhibiting MMR deficiency and high MSI (MSI-H) typically display a high tumor mutational burden and increased neoantigen load, which renders them more immunogenic. Recently, the MSI/dMMR condition has been shown to be a promising prognostic and predictive factor for discriminating both patients who could benefit from immunotherapy [59] and those who would have a low risk of recurrence [60,61]. Immune Checkpoint Inhibitor (ICI) drugs, like pembrolizumab, have shown better results and fewer side effects than chemotherapy, increasing the interest in this field [62,63,64]. In the literature, multiple studies indicate ctDNA as an eligible analyte in MSI status investigations [65], matching its results with those deriving from tissue biopsies [66], and in monitoring the response to immunotherapy as well [67,68,69]. In the present study, we even tried to find a possible correlation between EV cluster signatures and patients’ MMR status, comparing MMR-deficient and MMR-proficient groups. Unfortunately, no significant association was found, although it would be worth extending this study to a larger cohort of patients. Actually, the main limitation of this study is represented by the restricted number of samples, as among the 33 enrolled patients, only 6 patients displayed MMR deficiency. This could partially justify the absence of significant insights from the analysis. A further limitation of the present study is that MSI status was not available for the enrolled patients. Instead, MMR characterization was systematically performed within the diagnostic–therapeutic pathway, which currently represents a clinically accepted surrogate for therapeutic decision-making.

The strength of our research is the approach we propose by using an innovative and potentially translational technology able to quantify blood-derived EV-bounded proteins, both internal and surface, to predict disease relapse in CRC patients. Further studies could be undertaken using such innovative technology to reveal other EV-bounded biomarkers in different tumor contests, for diagnostic and prognostic intents.

## 5. Conclusions

Although the mortality rate of CRC is steadily declining, particularly in countries with a high HDI, thanks to advances in prevention, early detection, molecular stratification, the management of disease progression, and both pharmacological and radiological treatments, CRC recurrence remains a significant clinical challenge. A substantial proportion of patients still experience disease relapse within five years following curative surgery. In light of this, current clinical guidelines suggest a closer follow-up even though reliable cornerstones are lacking.

To this point of view, discovering new biomarkers to follow the clinical course of patients in a non-invasive manner is mandatory, especially for adjusting the adopted therapy and preventing acquired resistance or for anticipating relapse phenomena. Liquid biopsy offers an extraordinary opportunity, and several research groups have shown the potential of extracellular vesicles, among others, to act as promising biomarkers.

In this paper, we propose a signature of sEV-bounded protein markers, specifically IL-6/CD81, IL-6/Cyclin D1, and the combination of all three these markers, to predict through liquid biopsy the potential relapse of patients who already experienced CRC conditions. Secondly, we prove the correlation of some sEV protein combinations with CRC stage and lymph node status. Among these biomarkers, the combinations IL-6/CD81 and IL-6/Cyclin D1/CD81 showed the strongest prognostic performance. In addition, EpCAM, either alone or in combination with PD-L1, yielded statistically significant (or borderline significant) associations with lymph node status. Moreover, differences in cluster counts between surface markers (CD81, EpCAM, PD-L1) and the intravesicular marker Cyclin D1 also correlated with tumor stage. Finally, this study, together with our recent research [43], offers an innovative experimental design which may inspire other groups to apply dSTORM, which has been already recommended by the latest MISEV 2023 guidelines as a state-of-the-art strategy to assess molecular heterogeneity at the single-vesicle level, in order to enforce its translatability into clinical practice. Although promising, in fact, other investigations are necessary to support this approach in order to make personalized medicine a closer aim.

## Figures and Tables

**Table 1:** List of antibodies used in WB.

Antibody	Dilution	Brand	Catalog Number
Mouse monoclonal anti-CD45	1:500	R&D Systems	MAB14303
Rabbit monoclonal anti-Tsg101	1:1000	Abcam	ab125011
Mouse monoclonal anti-β-Actin	1:4000	Sigma Aldrich	A1978
Mouse monoclonal anti-β-Tubulin	1:1000	Cell Signaling technology	86298
Mouse monoclonal anti-CD63	1:1000	Invitrogen	10628D
Mouse monoclonal anti-CD9	1:1000	Invitrogen	10626D
Goat anti-mouse IgG (H + L) secondary antibody, Alexa Fluor Plus 555	1:5000	Invitrogen	A32727
Goat anti-mouse IgG (H + L) secondary antibody, Alexa Fluor Plus 488	1:5000	Invitrogen	A32766
Goat anti-rabbit IgG (H + L) secondary antibody, Alexa Fluor Plus 800	1:5000	Invitrogen	A32808
HRP-conjugated anti-rabbit secondary antibody	1:10,000	Invitrogen	31460
HRP-conjugated anti-mouse secondary antibody	1:10,000	Dako	P0447

**Table 2:** CRC patients’ clinical characteristics. Data are presented as mean with ranges for age and total with percentages in parentheses for remaining parameters. Abbreviations: y.o.: years-old; MMR: mismatch repair.

AGE (y.o.)	67.4 (37–82)
GENDER	
Males	18 (55%)
Females	15 (45%)
STAGING	
IIA	17 (52%)
IIB	3 (9%)
IIC	2 (6%)
IIIB	8 (24%)
IIIC	3 (9%)
TUMOR SIZE (T STATUS)	
T3	26 (79%)
T4	6 (18%)
Not Available	1 (3%)
LYMPH NODE STATUS	
N0	21 (64%)
N1	9 (27%)
N2	2 (6%)
Not Available	1 (3%)
MMR STATUS	
Proficient	26 (79%)
Deficient	5 (15%)
Not Available	2 (6%)
RECURRENCE	
No	27 (85%)
Yes	6 (15%)

**Table 3:** Contingency tables and performance metrics for intravesicular and surface targets using specific cutoff values.

	NO	YES	Sensitivity	Specifcity	Accuracy
IL6,CyclinD1,CD81 ≥ 7.62	5	4	0.81	0.67	0.79
IL6,CyelinD1,CD81 < 7.62	22	2			
IL6,CD81 ≥ 5.21	8	4	0.70	0.67	0.70
IL6,CD81 ≤ 5.21	19	2			
IL6,CyclinD1 ≥ 9.56	5	4	0.81	0.67	0.79
IL6,CyclinD1 < 9.56	22	2			
CD81,CyclinD1 ≥ 6.13	8	4	0.70	0.67	0.70
CD81,CyclinD1 < 6.13	19	2			
IL6 ≥ 7.55	12	3	0.56	0.50	0.55
IL6 < 7.55	15	3			
CD81 ≥ 4.91	8	4	0.70	0.67	0.70
CD81 < 4.91	19	2			
CyclinD1 ≥ 9.06	13	3	0.52	0.50	0.52
CyclinD1 < 9.06	14	3			

## Data Availability

All data are available as Supporting Materials, except for the raw super-resolution image data, which may be provided upon request due to size constraints.

## Supplementary Materials

The following supporting information can be downloaded at https://www.mdpi.com/article/10.3390/biom15091307/s1, Figure S1: Original Western Blot images; Figure S2: Intravesicular and surface markers without statistically significant differences between recurrence conditions; Figure S3: ROC curves for surface markers that are not statistically significant; Figure S4: Surface marker cluster count analysis between negative (N0) and positive (N+) lymph node status; Figure S5: Intravesicular and intravesicular/surface marker cluster count analysis between negative (N0) and positive (N+) lymph node status; Figure S6: Cluster counts grouped by lymph node status for intravesicular and surface markers; Figure S7: Cluster counts across tumor stages for intravesicular and surface markers (not statistically significant markers); Figure S8: Cluster counts across tumor stages II and III for surface markers; Figure S9: Cluster counts across tumor stages II and III for intravesicular and intravesicular/surface markers; Figure S10: Cluster counts for mismatch repair status for intravesicular and surface markers (not statistically significant markers); Table S1: Summary table of NTA performed on five representative sEV samples isolated from CRC patients; Table S2: Contingency table with cutoff values and performance metrics for markers: EpCAM, PD-L1, and CD81; EpCAM and CD81; EpCAM and PD-L1; CD81 and PD-L1; EpCAM; PD-L1.

## Abbreviations

The following abbreviations are used in this manuscript:

AF	Alexa Fluor dye
AUC	Area under the curve
CF	Cyanine-based far red fluorescent dye
CRC	Colorectal cancer
ctDNA	Circulating tumor DNA
CTCs	Circulating tumor cells
dMMR	Deficient mismatch repair
dSTORM	Direct stochastic optical reconstruction microscopy
EpCAM	Epithelial cell adhesion molecule
ESCRT	Endosomal sorting complexes required for transport
EVs	Extracellular vesicles
HDI	Human development index
ICI	Immune checkpoint inhibitor
IHC	Immunohistochemistry
IL-6	Interleukin-6
KRAS	Kirsten rat sarcoma virus
MMR	Mismatch repair
MSI	Microsatellite instability
NRAS	Neuroblastoma RAS oncogene
NTA	Nanoparticle tracking analysis
PD-L1	Programmed cell death 1 ligand 1
ROC	Receiver operating characteristic
RT	Room temperature
SEM	Scanning electron microscopy
UC	Ultracentrifugation
WB	Western blot
