# Supplementary material for: Single-Vesicle Molecular Profiling by dSTORM Imaging in a Liquid Biopsy Assay Predicts Early Relapse in Colorectal Cancer
Source: Biomolecules. 2025 Sep 11;15(9):1307. doi: 10.3390/biom15091307 (PMC12467539; doi:10.3390/biom15091307)
Supplement: Supplementary file 1 [file biomolecules-15-01307-s001.zip › biomolecules-3800364-Supplementary.pdf]

Sample	Modes	Mean	Standard Deviation	D10	D50	D90	Concentration [particles/mL]	Total particles	Valid tracks	Total Tracks	% valid tracks
1	46.5	106	176	36	66	218	3.34E+11	1.34E+11	11077	55817	20%
2	46.5	85	107	36	60	150	4.97E+11	1.99E+11	14806	84588	18%
3	44.5	75	57	35	59	129	6.24E+11	2.50E+11	14960	80121	19%
4	45.5	97	91	38	70	175	3.65E+11	1.46E+11	14054	73517	19%
5	50.5	78	60	36	61	135	4.60E+11	1.84E+11	18741	100053	19%

Table S1: Summary table of NTA Analysis performed on five representative sEV samples isolated from CRC patients.

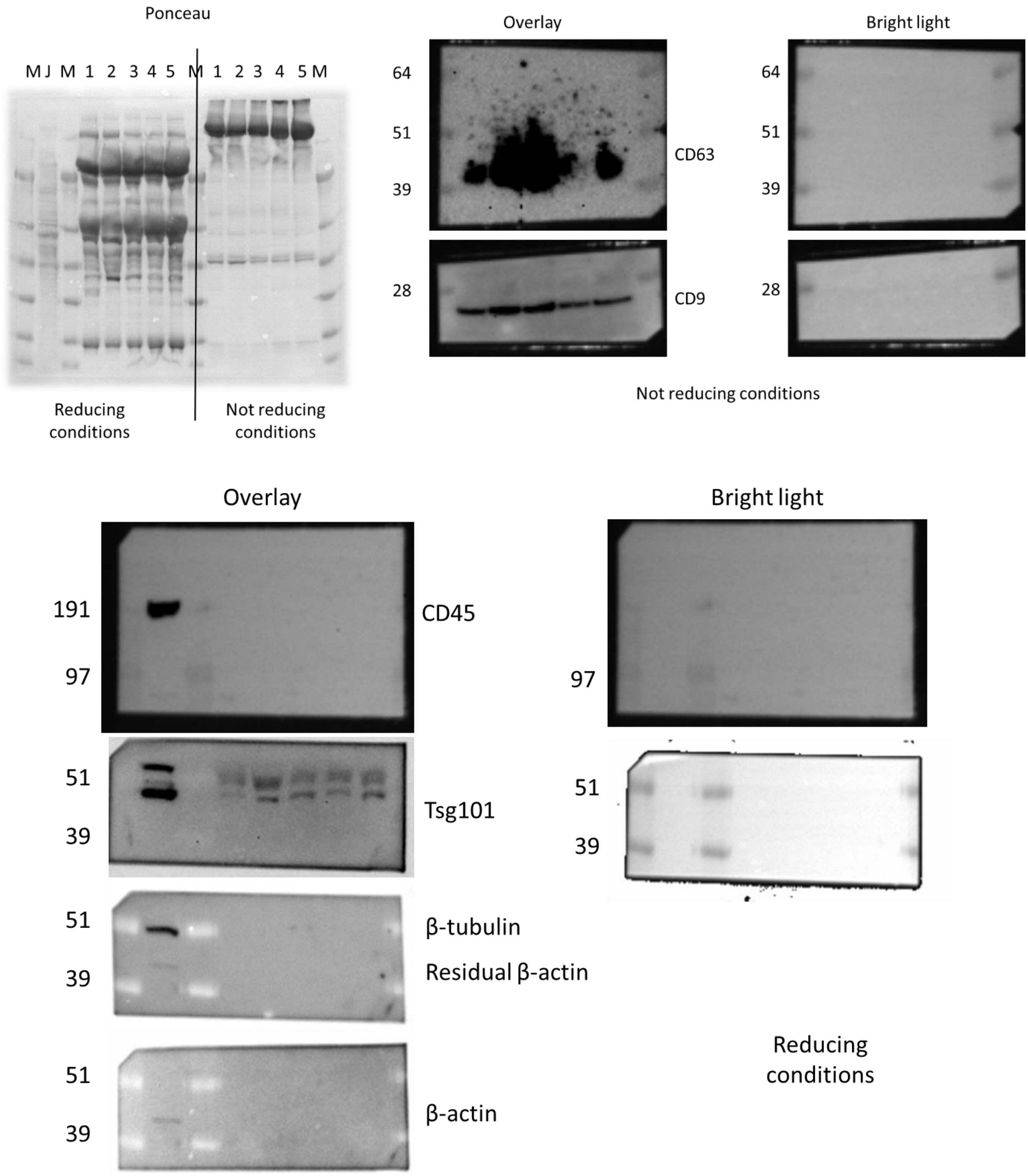

Figure S1. Original Western Blot images.

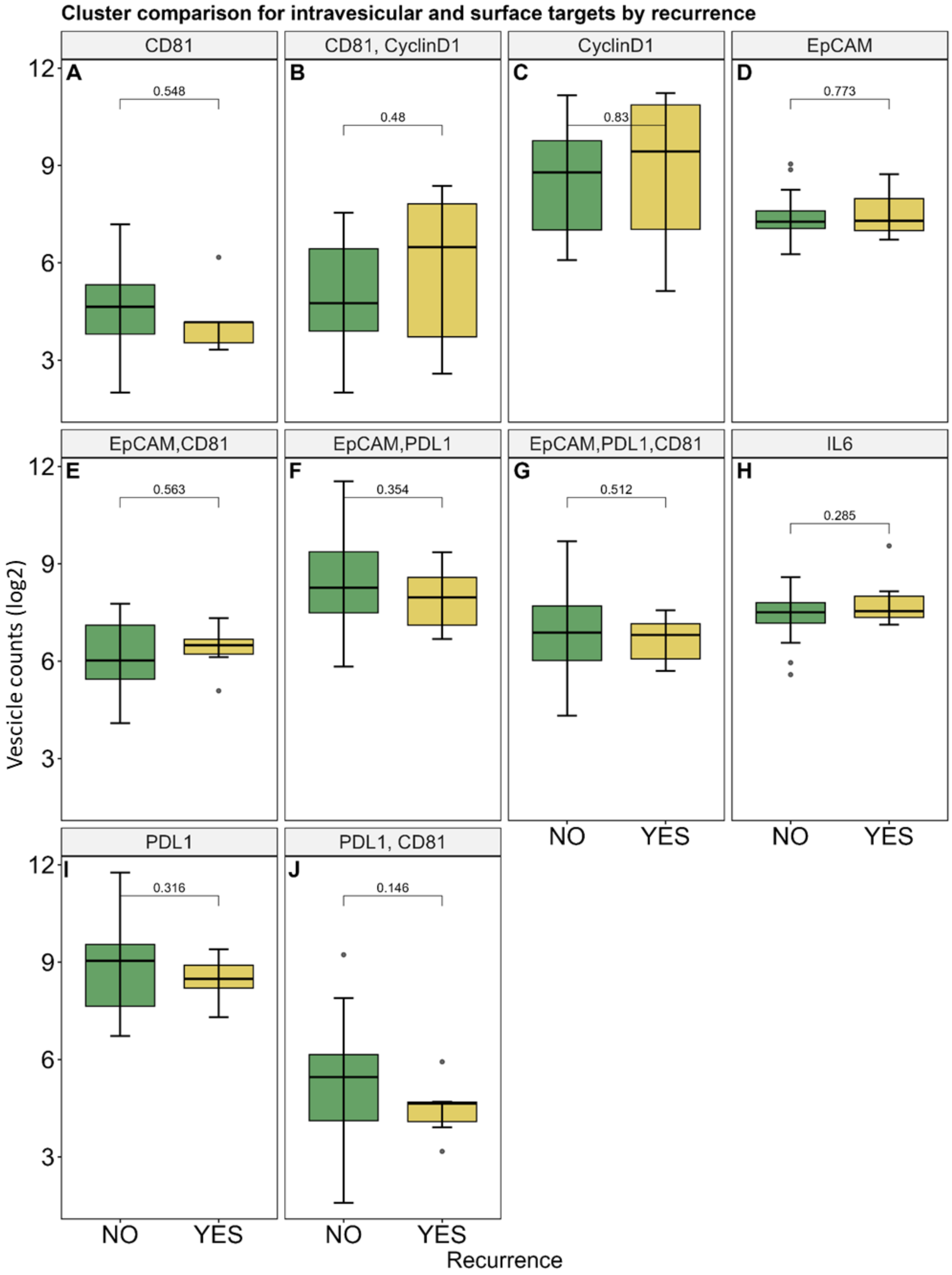

Figure S2. Intravesicular and surface markers without statistically significant differences between recurrence conditions.

ROC curves by intravescicular and surface target to discriminate recurrence condition

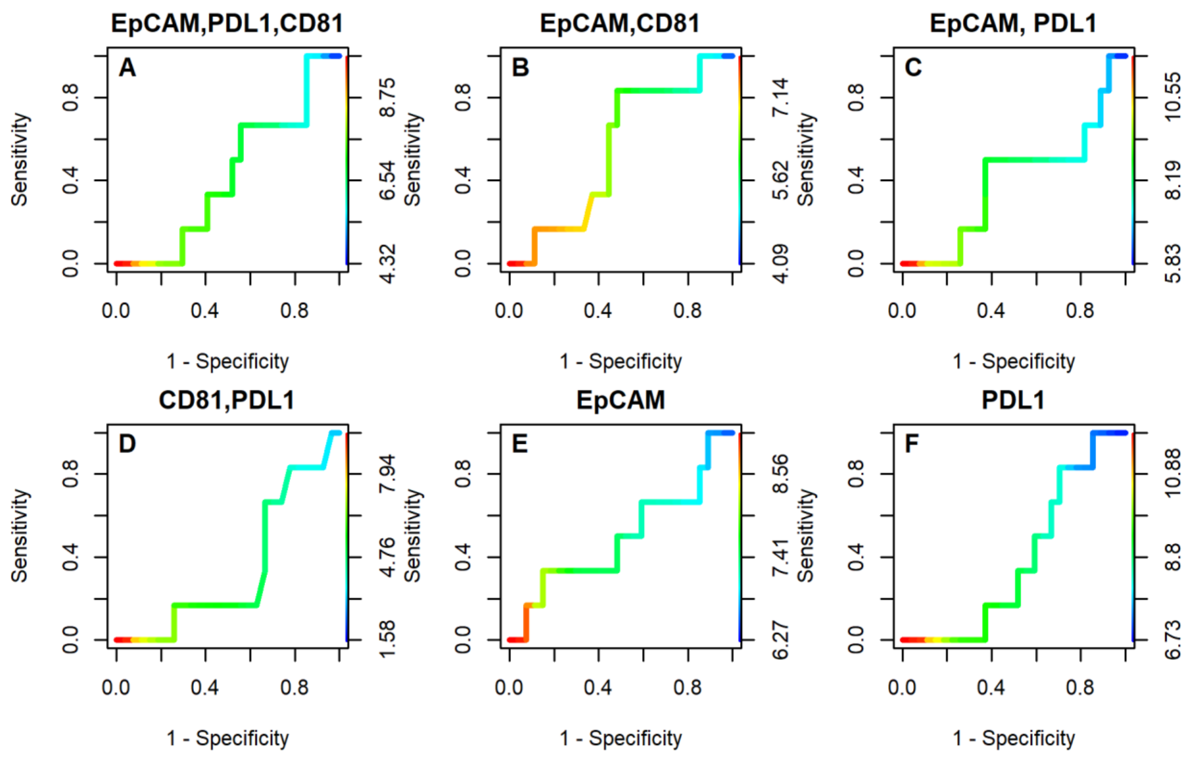

Figure S3. ROC curves for surface markers that were not statistically significant, demonstrating low discriminatory power. Specifically: EpCAM , PDL1 and CD81 shows an AUC of 0.42 (Fig. S5A); EpCAM and CD81, an AUC of 0.55 (Fig. S5B); EpCAM and PDL1, an AUC of 0.40 (Fig. S5C); CD81 and PDL1, an AUC of 0.34 (Fig. S5D); EpCAM, an AUC of 0.49 (Fig. S5E); PDL1, an AUC of 0.38 (Fig. S5F).

	NO	YES	Sensitivity	Specificity	Accuracy
EpCAM, PDL1,CD81≥ 6.88	14	2	0.48	0.33	0.45
EpCAM, PDL1,CD81 <6.88	13	4			
EpCAM,CD81 ≥ 6.52	12	2	0.56	0.33	0.52
EpCAM,CD81 < 6.52	15	4			
EpCAM,PDL1 ≥ 8.31	13	3	0.52	0.50	0.52
EpCAM,PDL1 < 8.31	14	3			
CD81,PDL1≥ 4.95	17	1	0.37	0.17	0.33
CD81,PDL1 < 4.95	10	5			
EpCAM ≥ 7.41	13	3	0.52	0.50	0.52
EpCAM < 7.41	14	3			
PDL1 ≥ 8.77	16	2	0.41	0.33	0.39
PDL1 < 8.77	11	4			

Table S2: Contingency table with cutoff values and performance metrics for markers: EpCAM, PDL1, CD81; EpCAM, CD81; EpCAM, PDL1; CD81, PDL1; EpCAM; PDL1.

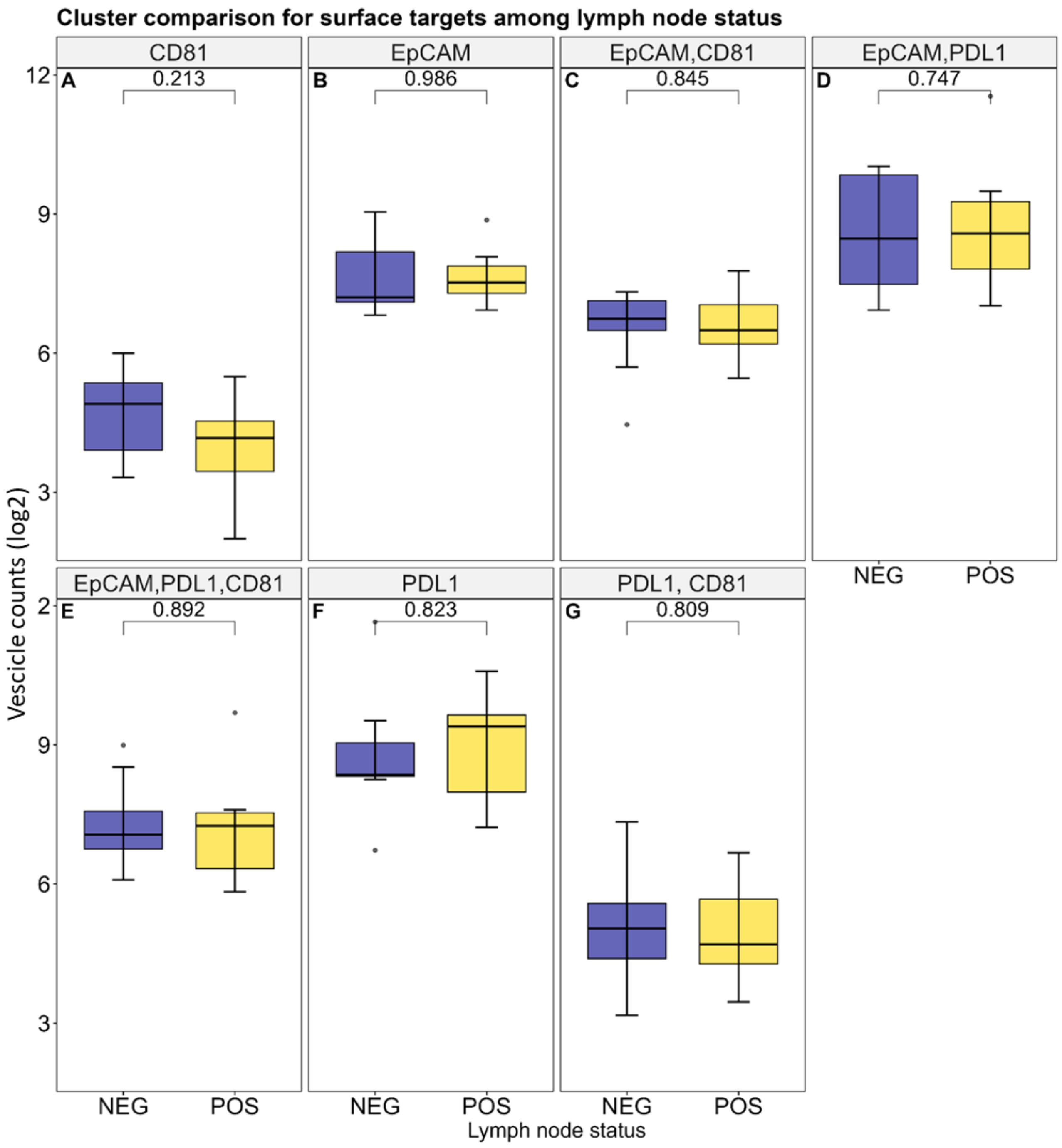

Figure S4. Surface markers cluster counts analysis between negative (N0) and positive (N+) lymph node status.

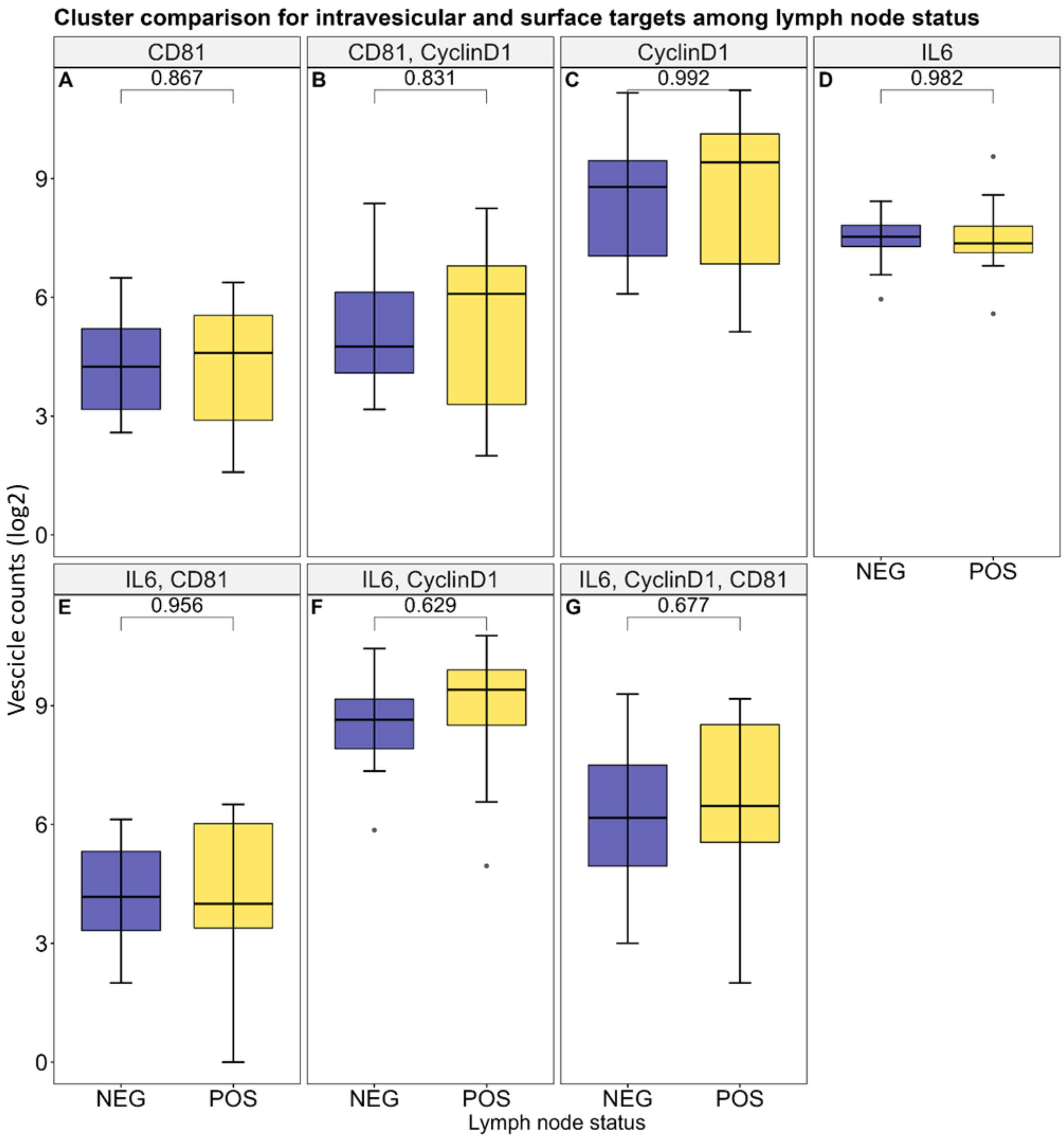

Figure S5. Intravesicular and intravesicular/surface markers cluster counts analysis between negative (N0) and positive (N+) lymph node status.

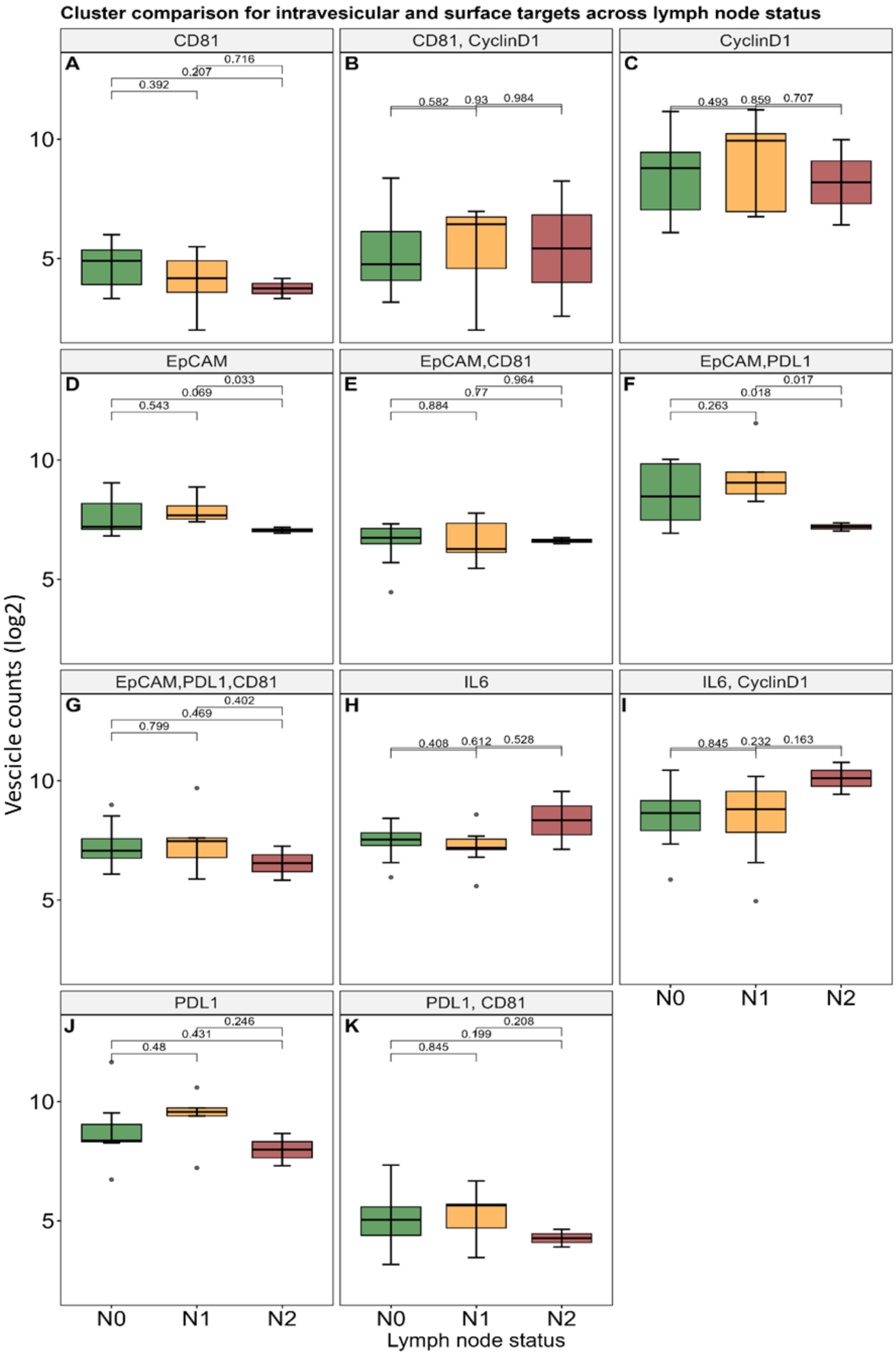

Figure S6. Cluster counts grouped by lymph node status for intravesicular and surface markers. EpCAM (Fig. S2D) shows statistically significant difference ($p < 0.05$) between N0 and N1 status, as well as between N1 and N2 status. In Fig. S2F, EpCAM and PDL1 show significantly lower values in N2 status compared to N0 and N1.

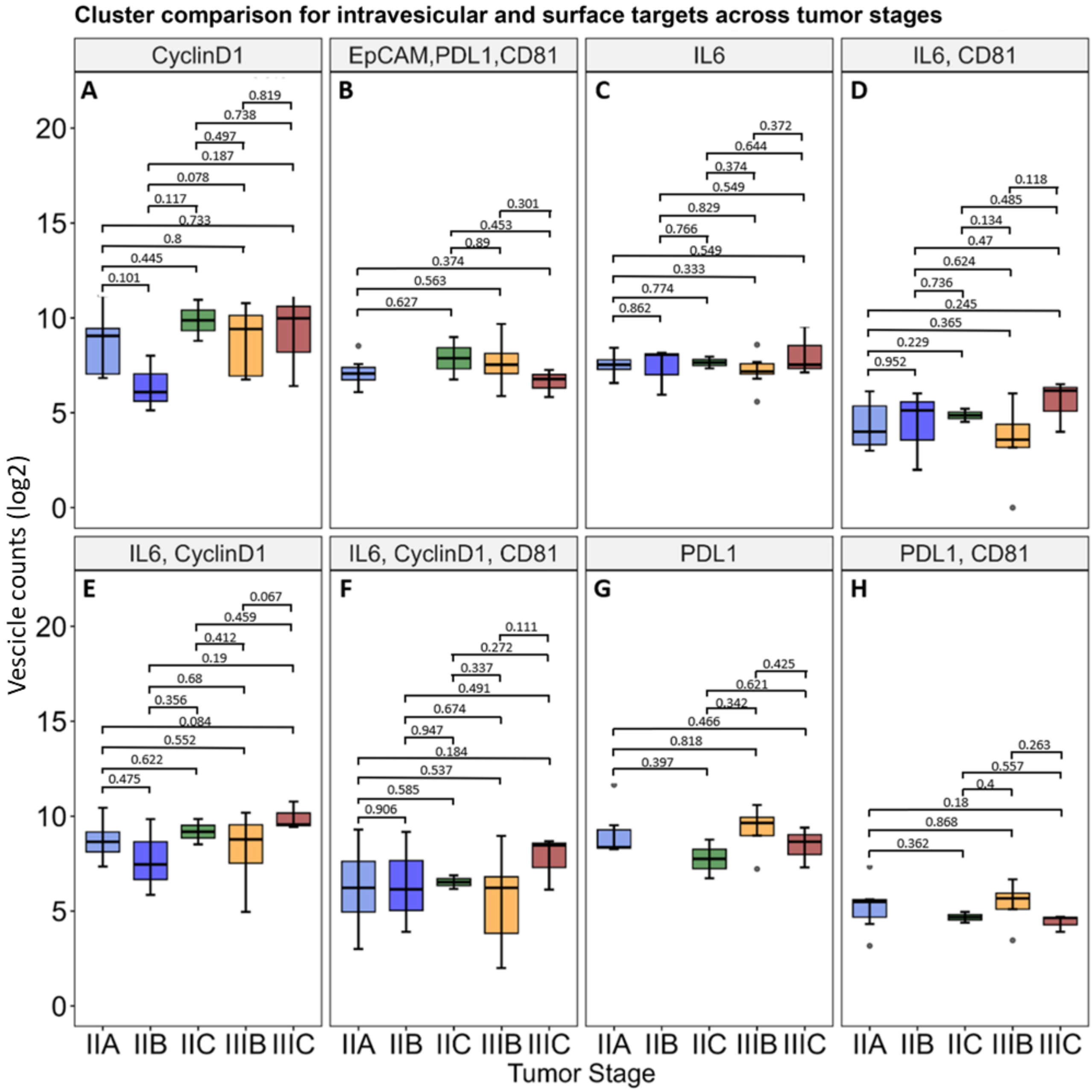

Figure S7. Cluster counts across tumor stages for intravesicular and surface markers (not statistically significant markers).

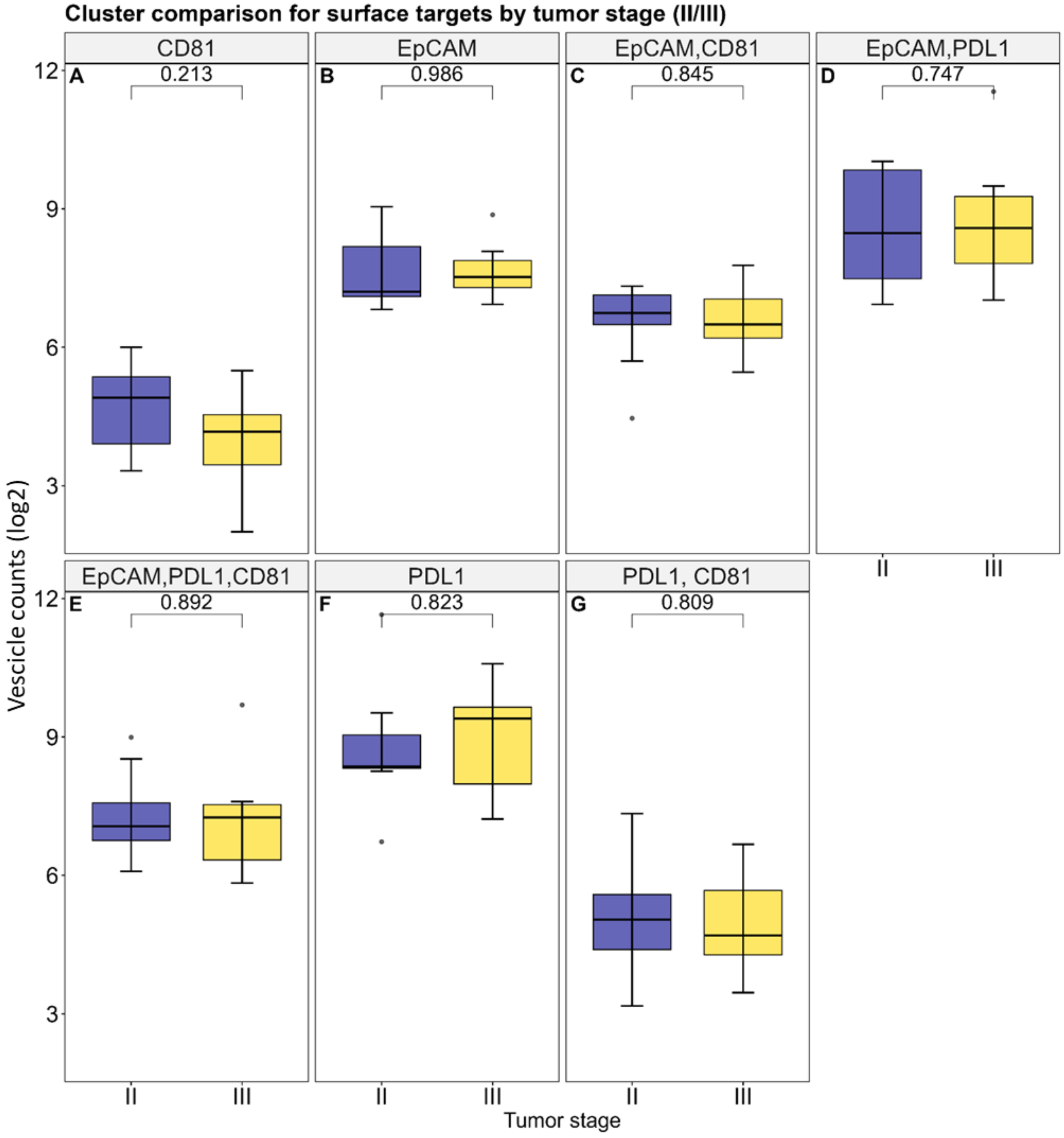

Figure S8. Cluster counts across tumor stages II and III for surface markers.

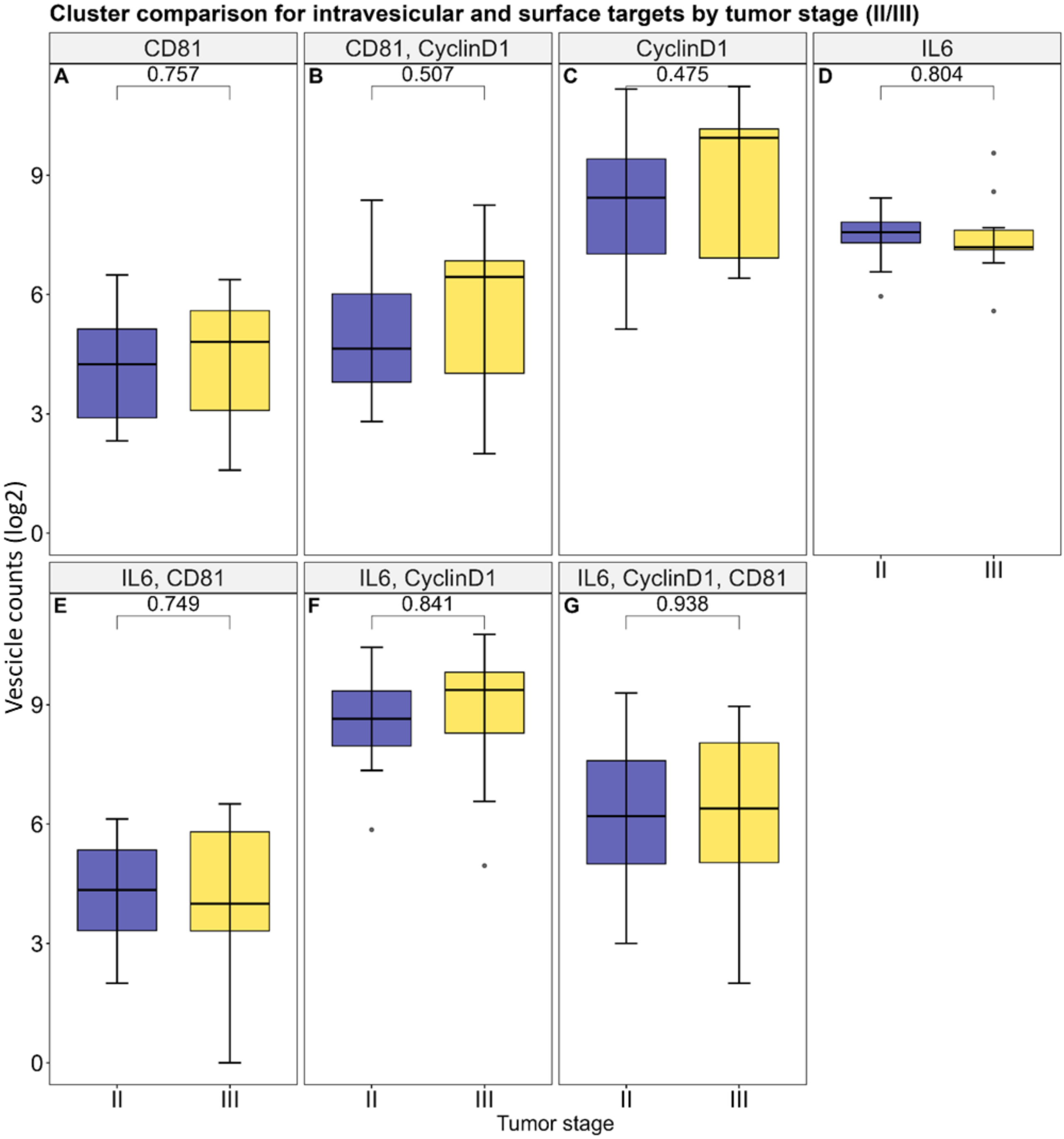

Figure S9. Cluster counts across tumor stages II and III for intravesicular and intravesicular/surface markers.

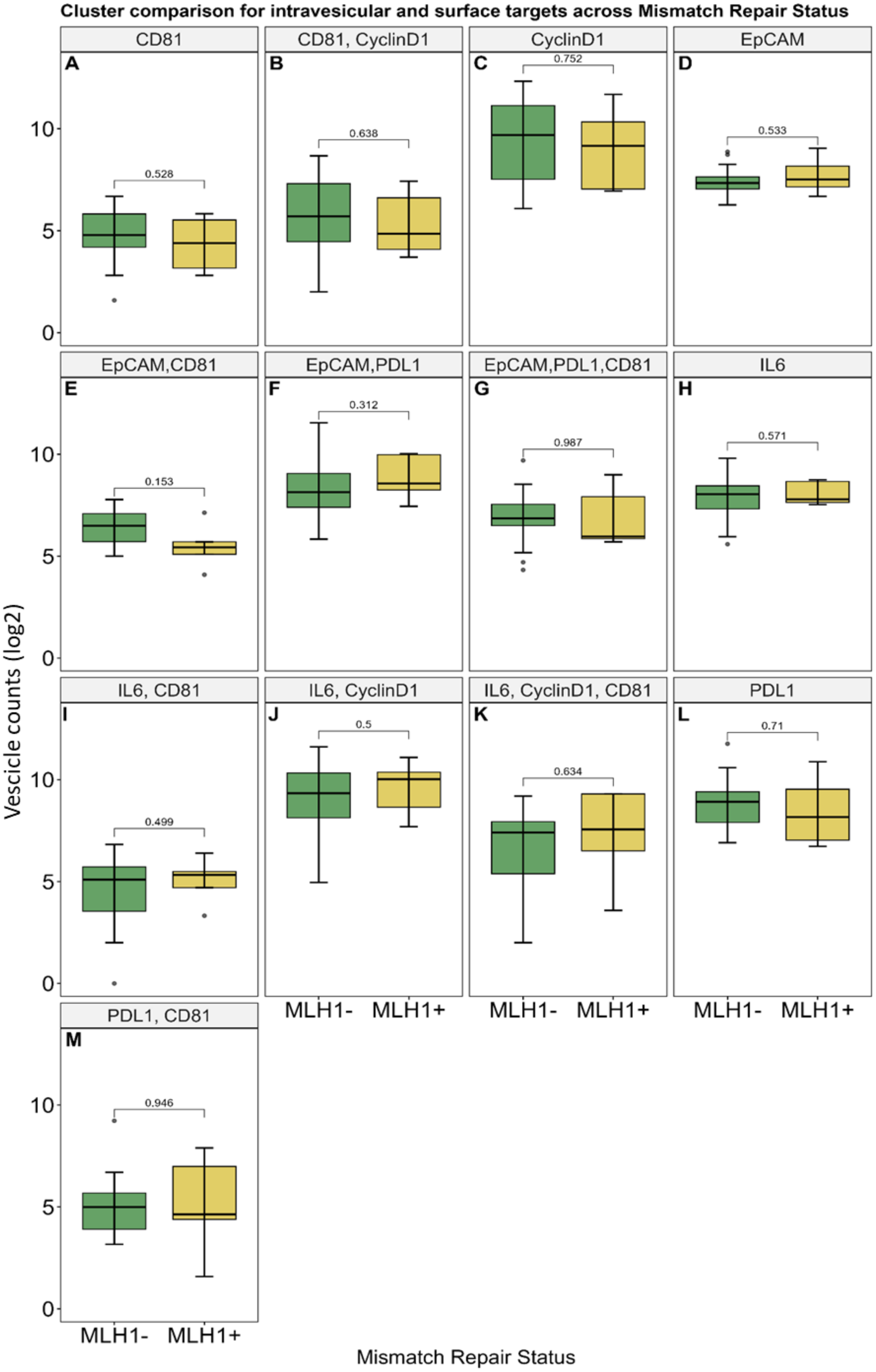

Figure S10. Cluster counts for Mismatch Repair status for intravesicular and surface markers (not statistically significant markers).
